# Analysis of EPID Transmission Fluence Maps Using Machine Learning Models and CNN for Identifying Position Errors in the Treatment of GO Patients

**DOI:** 10.3389/fonc.2021.721591

**Published:** 2021-09-14

**Authors:** Guyu Dai, Xiangbin Zhang, Wenjie Liu, Zhibin Li, Guangyu Wang, Yaxin Liu, Qing Xiao, Lian Duan, Jing Li, Xinyu Song, Guangjun Li, Sen Bai

**Affiliations:** ^1^Department of Radiation Oncology, Cancer Center and State Key Laboratory of Biotherapy, West China Hospital, Sichuan University, Chengdu, China; ^2^Machine Intelligence Laboratory, College of Computer Science, Sichuan University, Chengdu, China

**Keywords:** EPID transmission fluence, radiomics, SSIM, machine learning, CNN

## Abstract

**Purpose:**

To find a suitable method for analyzing electronic portal imaging device (EPID) transmission fluence maps for the identification of position errors in the *in vivo* dose monitoring of patients with Graves’ ophthalmopathy (GO).

**Methods:**

Position errors combining 0-, 2-, and 4-mm errors in the left-right (LR), anterior-posterior (AP), and superior-inferior (SI) directions in the delivery of 40 GO patient radiotherapy plans to a human head phantom were simulated and EPID transmission fluence maps were acquired. Dose difference (DD) and structural similarity (SSIM) maps were calculated to quantify changes in the fluence maps. Three types of machine learning (ML) models that utilize radiomics features of the DD maps (ML 1 models), features of the SSIM maps (ML 2 models), and features of both DD and SSIM maps (ML 3 models) as inputs were used to perform three types of position error classification, namely a binary classification of the isocenter error (type 1), three binary classifications of LR, SI, and AP direction errors (type 2), and an eight-element classification of the combined LR, SI, and AP direction errors (type 3). Convolutional neural network (CNN) was also used to classify position errors using the DD and SSIM maps as input.

**Results:**

The best-performing ML 1 model was XGBoost, which achieved accuracies of 0.889, 0.755, 0.778, 0.833, and 0.532 in the type 1, type 2-LR, type 2-AP, type 2-SI, and type 3 classification, respectively. The best ML 2 model was XGBoost, which achieved accuracies of 0.856, 0.731, 0.736, 0.949, and 0.491, respectively. The best ML 3 model was linear discriminant classifier (LDC), which achieved accuracies of 0.903, 0.792, 0.870, 0.931, and 0.671, respectively. The CNN achieved classification accuracies of 0.925, 0.833, 0.875, 0.949, and 0.689, respectively.

**Conclusion:**

ML models and CNN using combined DD and SSIM maps can analyze EPID transmission fluence maps to identify position errors in the treatment of GO patients. Further studies with large sample sizes are needed to improve the accuracy of CNN.

## Introduction

As a treatment method for Graves’ ophthalmopathy (GO), an eye disease related to autoimmune thyroid disease (1), radiotherapy can be applied with satisfactory control while producing relatively slight post-radiotherapeutic complications (2). The organs-at-risk (OARs) around the target volumes of GO include the lens, optic nerves, and similar tissues. To enable the delivery of conformal and uniform doses to these target volumes while reducing the dose received by normal tissue, intensity-modulated radiotherapy (IMRT) and volumetric modulated arc therapy (VMAT) are often used for GO patients (1–3) because these approaches generate a steep dose gradient between the target volume and OARs (4, 5). However, this implies that errors during treatment, such as position errors, have a significant impact on the treatment results (6). Cone beam computed tomography (CBCT), which is often used for correcting position errors before treatment, introduces additional radiation doses to patients (7); furthermore, intra-fractional movement is still present after pre-treatment CBCT scanning (8–10). As a method for monitoring treatment, *in vivo* dosimetry for obtaining information on the doses delivered to patients has significant potential.

Currently, amorphous silicon electronic portal imaging devices (a-Si EPIDs), which have high spatial resolutions, large two-dimensional arrays, and approximately linear dose responses (11–13), are commonly used in clinical *in vivo* dosimetry (14–18). Although gamma pass rate threshold-based EPID *in vivo* dosimetry can be used to monitor treatment through single pass rate values (16–19), research on EPID dosimetry by Hsieh Emmelyn S et al. (20) has revealed that, under 3%/3 mm and 95% pass rate threshold criteria, position errors greater than 2 cm can be detected, which is unsatisfactory. Meanwhile, gamma pass rate threshold-based dosimetry compresses 2D image information and therefore causes information loss (21). In addition, using gamma pass rate, errors can be detected; however, their direction cannot be detected. Thus, a tool for more comprehensive detection of errors and their direction is required.

Structural similarity (SSIM), which can measure the similarities between pairs of images based on the image luminance, contrast, and structural similarity, has been used for dose distribution error detection (22). Meanwhile, research showed that linac mechanical errors can be detected by combining dose difference (DD) maps with SSIM maps to analyze EPID fluence (23). However, DD maps and SSIM maps cannot be analyzed manually to detect errors.

The collection of methods for extracting information such as shape, grayscale, and texture contained in digital medical images into high-dimensional data is known as radiomics (24, 25). Because radiomics information contains significant quantities of data that are difficult to process manually, radiomics is often combined with artificial intelligence for diagnosis, treatment selection, and prediction (26–29). In several recent studies, radiomics has been combined with machine learning (ML) for conducting EPID fluence-based dose verification (21, 23, 30).

Convolutional neural networks (CNNs) can effectively perform image-related tasks by analyzing images at different scales using convolutional layers to extract useful information and generate final outputs (31). Accordingly, a number of researchers have proposed CNN-based patient-specific dose verification using dose maps as the CNN input (32–35).

This study aims to find a suitable method for analyzing EPID transmission fluence maps in the identification of position errors during the *in vivo* dose monitoring of GO patients. To this end, we measured EPID transmission fluence maps with and without position errors obtained from the treatment of a head phantom and used the maps without position errors as a baseline for calculating dose difference and structural similarity maps reflecting position errors. The radiomics features of these DD and SSIM maps were then combined with ML models to classify position errors. CNN was also established to classify position errors using the DD and SSIM maps as inputs, and the ML and CNN classification results were compared.

## Methods

### Position Error Simulation and EPID Transmission Fluence Map Measurement

**Figure 1:**
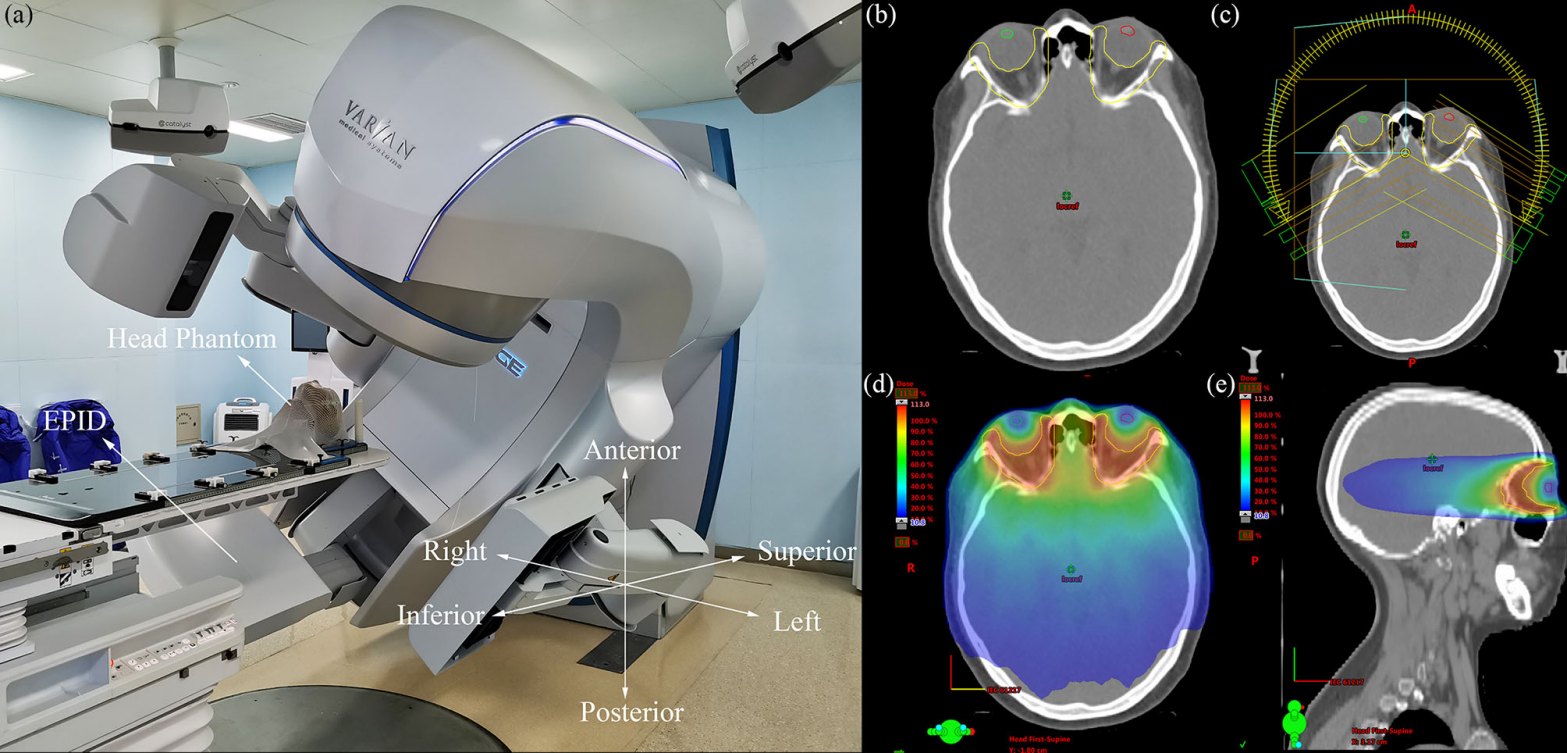
**(A)** Photograph of the measurement process, the coordinate system in the lower right corner indicates the direction of the position errors, **(B)** transverse plane of one GO patient, the yellow region of interest (ROI) is PTV, the green and red ROI are lens, **(C)** VMAT plan with two partial arcs rotating from 240 to 120° clockwise and from 120 to 240° counterclockwise, **(D)** the dose distribution of transverse plane, **(E)** the dose distribution of sagittal plane.

Forty VMAT plans of patients with GO who received radiotherapy in our department from November 2019 to October 2020 were selected, with a prescription dose of 20 Gy in 10 fractions. VMAT with two partial arcs rotating from 240 to 120° clockwise and from 120 to 240° counterclockwise was used to design the radiotherapy plans. To simulate clinical treatment, the plans were delivered to a head phantom (Chengdu Dosimetric Phantoms, Chengdu, China) on an EDGE linac (Varian Medical Systems, Palo Alto, CA) (**Figure 1** **A**). The beam transmission fluence maps were measured by an as1200 EPID (Varian Medical Systems, Palo Alto, CA) with 1190 × 1190 pixels and pixel size of 0.336 mm × 0.336 mm in dosimetry mode. For each patient, one measurement without position errors was conducted first to obtain a baseline transmission fluence map. Then, 27 fluence maps with position errors were measured while 27 possibilities of position errors (**Table 1**) combining 0-, 2-, and 4-mm left-right (LR), anterior-posterior (AP), and superior-inferior (SI) position errors were simulated by translating the head phantom along the LR, AP, and SI axis. The 0, 0, 0 error position was the same as the position where baseline maps were measured. Prior to measurement, the background and pixel response of the EPID were calibrated through a dark field and a flood field and the EPID dose was calibrated.

**Table 1:** Position errors combining 0-, 2-, and 4-mm errors in LR, AP, and SI directions and classification labels of different types of position errors for three types of classification tasks.

Direction	Serial number	Set-up errors	Isocenter errors (mm)	Classification Labels				
				Type 1	Type 2-LR	Type 2-SI	Type 2-AP	Type 3
None	1	Without errors (baseline)	/	/	/	/	/	/
	2	Without errors	0.00	1	1	1	1	1
Single Direction	3	LR 2mm	2.00	1	1	1	1	1
	4	LR 4mm	4.00	2	2	1	1	2
	5	SI 2mm	2.00	1	1	1	1	1
	6	SI 4mm	4.00	2	1	2	1	4
	7	AP 2mm	2.00	1	1	1	1	1
	8	AP 4mm	4.00	2	1	1	2	3
Double Directions	9	LR 2mm+AP 4mm	4.47	2	1	1	2	3
	10	LR 4mm +AP 4mm	5.66	2	2	1	2	5
	11	LR 4mm +AP 2mm	4.47	2	2	1	1	2
	12	LR 2mm +AP 2mm	2.83	1	1	1	1	1
	13	LR 2mm +SI 2mm	2.83	1	1	1	1	1
	14	LR 2mm +SI 4mm	4.47	2	1	2	1	4
	15	LR 4mm +SI 4mm	5.66	2	2	2	1	6
	16	LR 4mm +SI 2mm	4.47	2	2	1	1	2
	17	SI 2mm +AP 2mm	2.83	1	1	1	1	1
	18	SI 2mm +AP 4mm	4.47	2	1	1	2	3
	19	SI 4mm +AP 4mm	5.66	2	1	2	2	7
	20	SI 4mm +AP 2mm	4.47	2	1	2	1	4
Triple Directions	21	LR 2mm +SI 2mm+AP 2mm	3.46	2	1	1	1	1
	22	LR 2mm+SI 2mm+AP 4mm	4.90	2	1	1	2	3
	23	LR 2mm+SI 4mm+AP 4mm	6.00	2	1	2	2	7
	24	LR 2mm+SI 4mm+AP 2mm	4.90	2	1	2	1	4
	25	LR 4mm+SI 4mm+AP 2mm	6.00	2	2	2	1	6
	26	LR 4mm+SI 2mm+AP 2mm	4.90	2	2	1	1	2
	27	LR 4mm+SI 2mm+AP 4mm	6.00	2	2	1	2	5
	28	LR 4mm+SI 4mm+AP 4mm	6.93	2	2	2	2	8

LR, left-right; AP, anterior-posterior; SI, superior-inferior $Isocenter\;error=\sqrt{LR\;errors^{2}+SI\;errors^{2}+AP\;errors^{2}}$.

### Calculation of DD and SSIM Maps

The DD map *DD(x,y)* used in this study is defined as


(1) $$DD(x,y)=\left|V_{x}-V_{y}\right|,$$

where *V_x_* and *V_y_* are the pixel values at a given spatial location on the baseline fluence map and fluence map with position errors, respectively.

SSIM is an indicator used to measure the similarity of two images, with SSIM = 1 indicating that the two images are the same (36). We define SSIM as


(2) $$SSIM(x,y)={\left[l(x,y)\right]}^{\alpha }\cdot {\left[c(x,y)\right]}^{\beta }\cdot {\left[s(x,y)\right]}^{\gamma },$$

where *α*, *β*, and *γ* are constants to control the relative weight of the three components, and *l(x,y)*, *c(x,y)*, and *s(x,y)* are the luminance, contrast, and structure maps, respectively, which are given by


(3) $$l(x,y)-\frac{2\mu_{x}\mu_{y}+c_{1}}{\mu_{x}^{2}+\mu_{y}^{2}+c_{1}},$$

(4) $$c(x,y)=\frac{2\delta_{x}\delta_{y}+c_{1}}{\delta_{x}^{2}+\delta_{y}^{2}+c_{2}},$$

(5) $$s(x,y)=\frac{\delta_{xy}+c_{3}}{\delta_{x}\delta_{y}+c_{3}},$$

where *μ_x_* and where *μ_y_* are the local mean values of the pixels of images *x* and *y*, respectively, *δ_x_* and *δ_y_* are the local standard deviations of the pixel values of images *x* and *y*, respectively, and *δ_xy_* is the covariance of images *x* and *y*. In this study, a square window with a side length of 11 pixels was used as the local window to calculate *μ_x_*, *μ_y_* , *δ_x_*, *δ_y_*, and *δ_xy_*, the constants *C_1_ = (K_1_L)^2^*, *C_2_ = (K_2_L)^2^*, and *C_3_ = C_2_/2* were used to avoid zero denominators, the default values *K*_1_ = 0.01 and *K*_2_ = 0.03 were obtained from Wang (36), and *L* = 200 was selected to represent the fraction dose 200 cGy.

**Figure 2:**
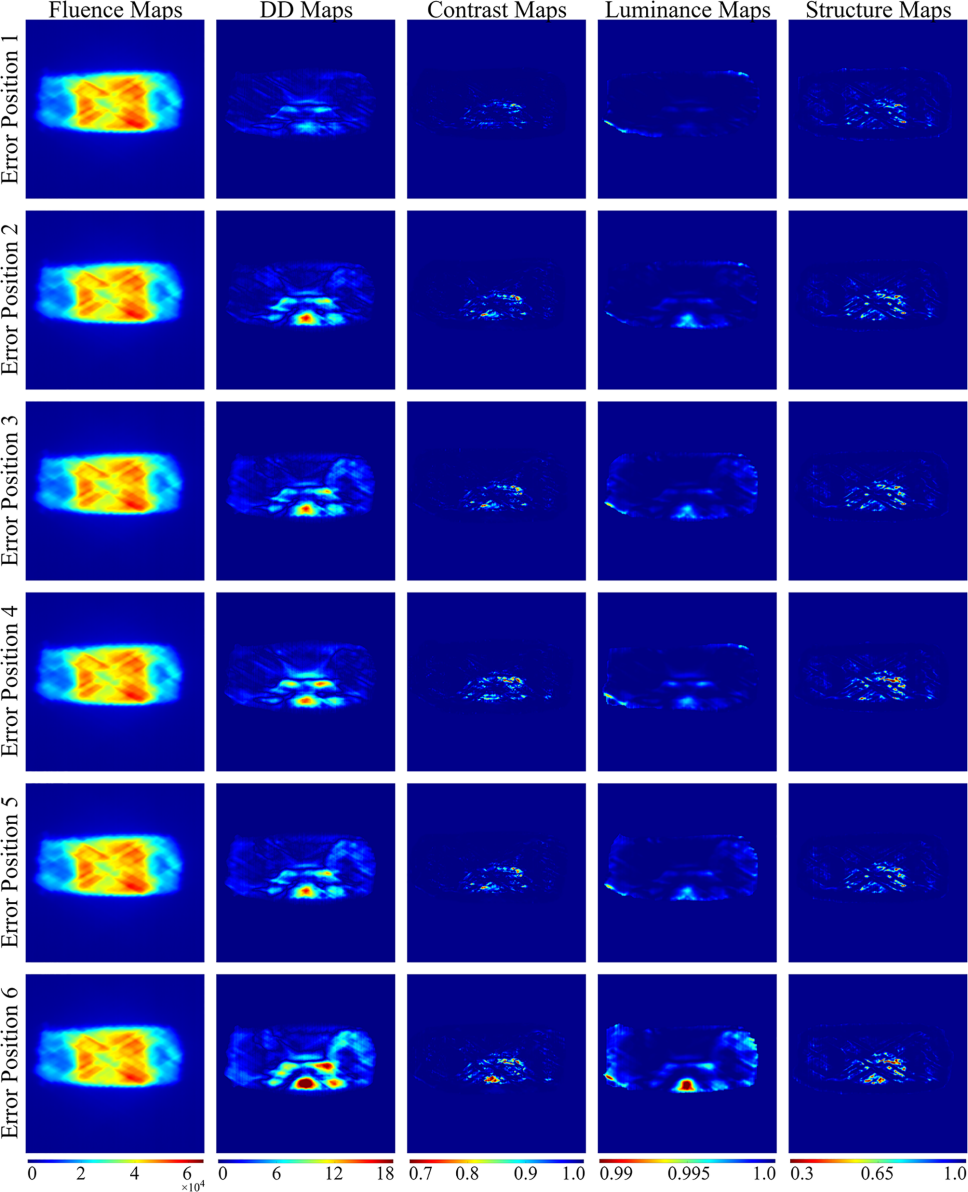
Overview of the fluence maps, calculated DD, contrast, luminance, and structure maps with six possibilities of error position for one patient. Error position 1 represents position with 2 mm SI errors; error position 2 represents position with 2 mm AP errors and 2 mm SI errors; error position 3 represents position with 2 mm LR errors, 2 mm AP errors, and 2 mm SI errors; error position 4 represents position with 4 mm SI errors; error position 5 represents position with 4 mm AP errors and 4 mm SI errors; error position 6 represents position with 4 mm LR errors, 4 mm AP errors, and 4 mm SI errors.

As shown in the lower left corner of **Figure 5**, to quantify EPID transmission fluence changes caused by the position error, DD maps and SSIM maps, including luminance, contrast, and structure maps, were calculated. The baseline fluence maps were compared to fluence maps with 27 possibilities of position errors; thus, for each patient, 27 sets of DD, luminance, contrast, and structure maps were calculated. Some of the measured fluence maps and calculated maps are shown in **Figure 2**.

### Position Error Classification

**Figure 3:**
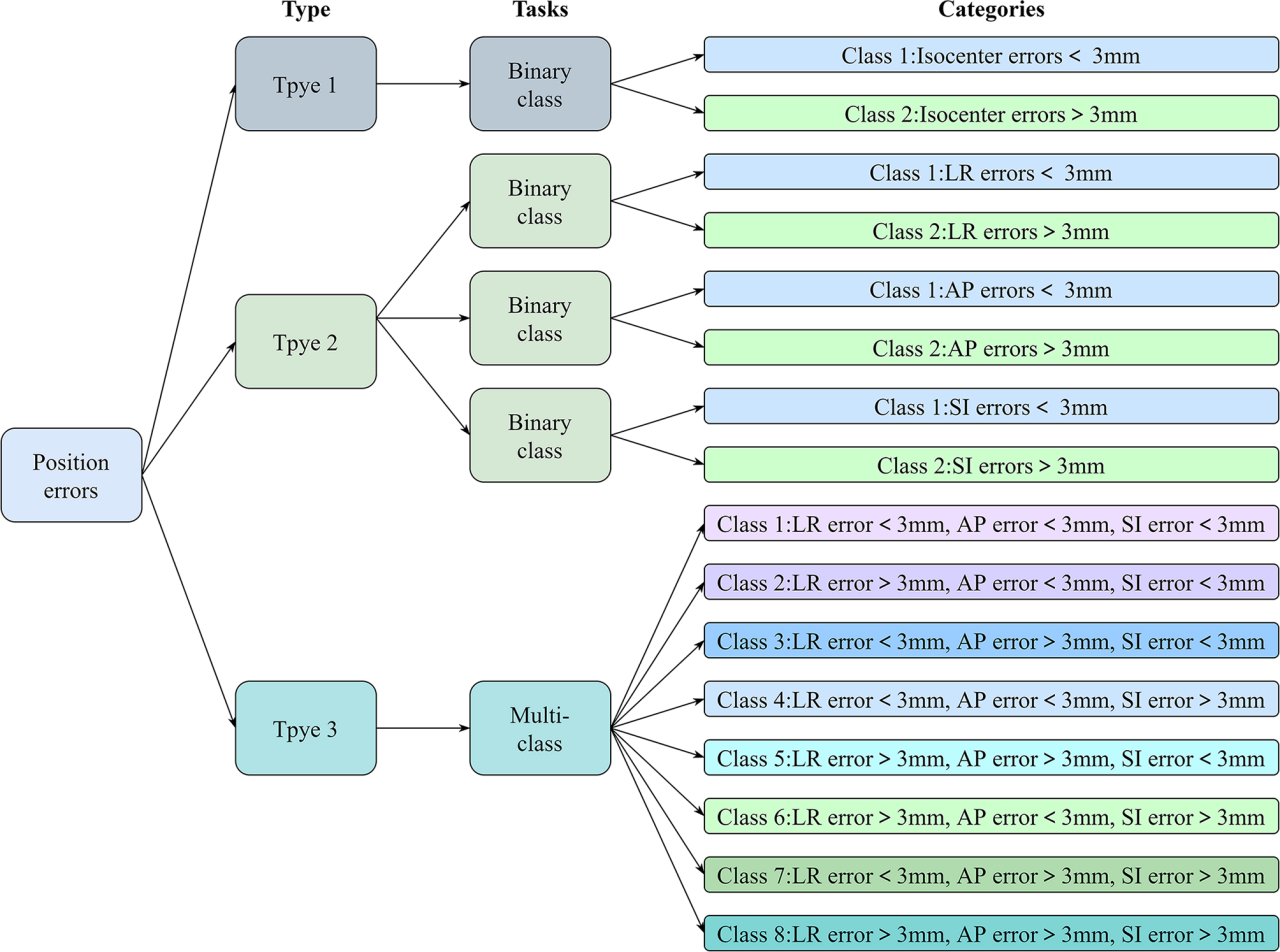
Overview of position error classification types and classes per type. LR, left-right; AP, anterior-posterior; SI, superior-inferior.

Three types of position error classifications were performed (**Figure 3**): type 1 classified errors into two classes based on whether the isocenter error was over 3 mm, and the isocenter is defined as


(6) $$Isocenter\;error=\sqrt{LR\;errors^{2}+SI\;errors^{2}+AP\;errors^{2}},$$

where *LR errors*, *SI errors*, and *AP errors* are the position errors in LR, SI and AP directions, respectively.

Type 2, which contained three classification tasks, classified errors into two classes depending on whether the error in the AP, SI, or LR direction was over 3 mm; type 3 combined the three types of classification methods of type 2 and classified errors into eight classes with the goal of predicting the direction from which any error larger than 3 mm originated.

### Machine Learning Method

**Figure 4:**
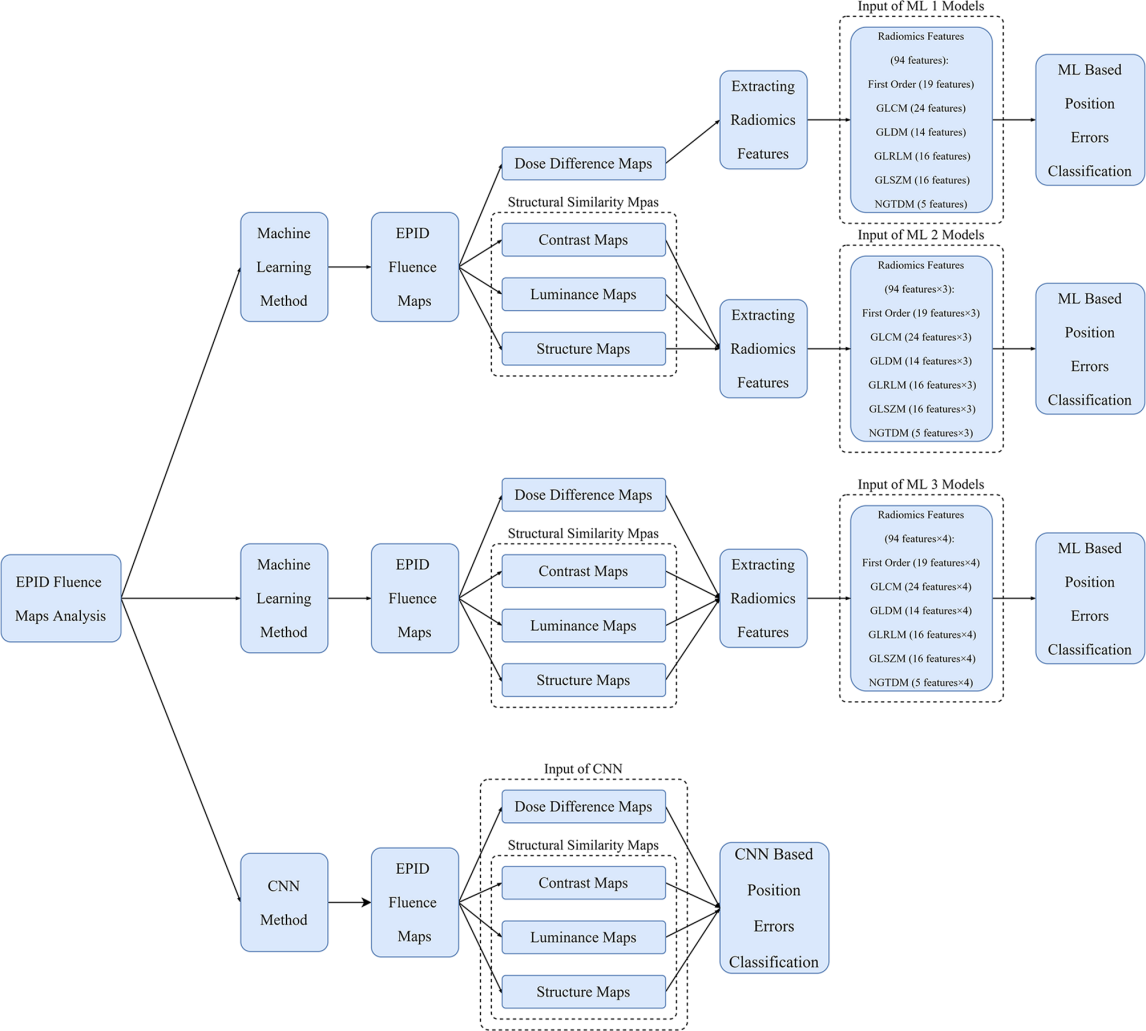
Overview of workflow of ML models and CNN for position error classification. The inputs of the ML 1 model are features of DD maps; those of the ML 2 model are features of SSIM maps; those of the ML 3 model are features of DD and SSIM maps; and those of the CNN are DD and SSIM maps.

The ML method used in the study is shown in **Figure 4**. The Python opensource library Pyradiomics (37) was used to extract radiomics features from the DD, luminance, contrast, and structure maps. Ninety-four types of features derived from six classes were extracted from the 512 × 512 center pixel matrix of each map, including first-order (19 features), gray level co-occurrence matrix (GLCM, 24 features), gray level dependence matrix (GLDM, 14 features), gray level run length matrix (GLRLM, 16 features), gray level size zone matrix (GLSZM, 16 features), and neighboring gray tone difference matrix (NGTDM, 5 features) features. Because of the constant shape of the fluence maps, the shape 2D (10 features) and shape 3D (17 features) features remained the same and were excluded. The classification models in the Python scikit-learn (38) and XGBoost (39) libraries, which included linear discriminant classifier (LDC), linear function kernel-supporting vector machine (linear-SVM), radial basis function kernel-supporting vector machine (RBF-SVM), K-nearest neighbor (KNN), and extreme gradient boosting (XGBoost) models, were used to classify the position errors.

**Figure 5:**
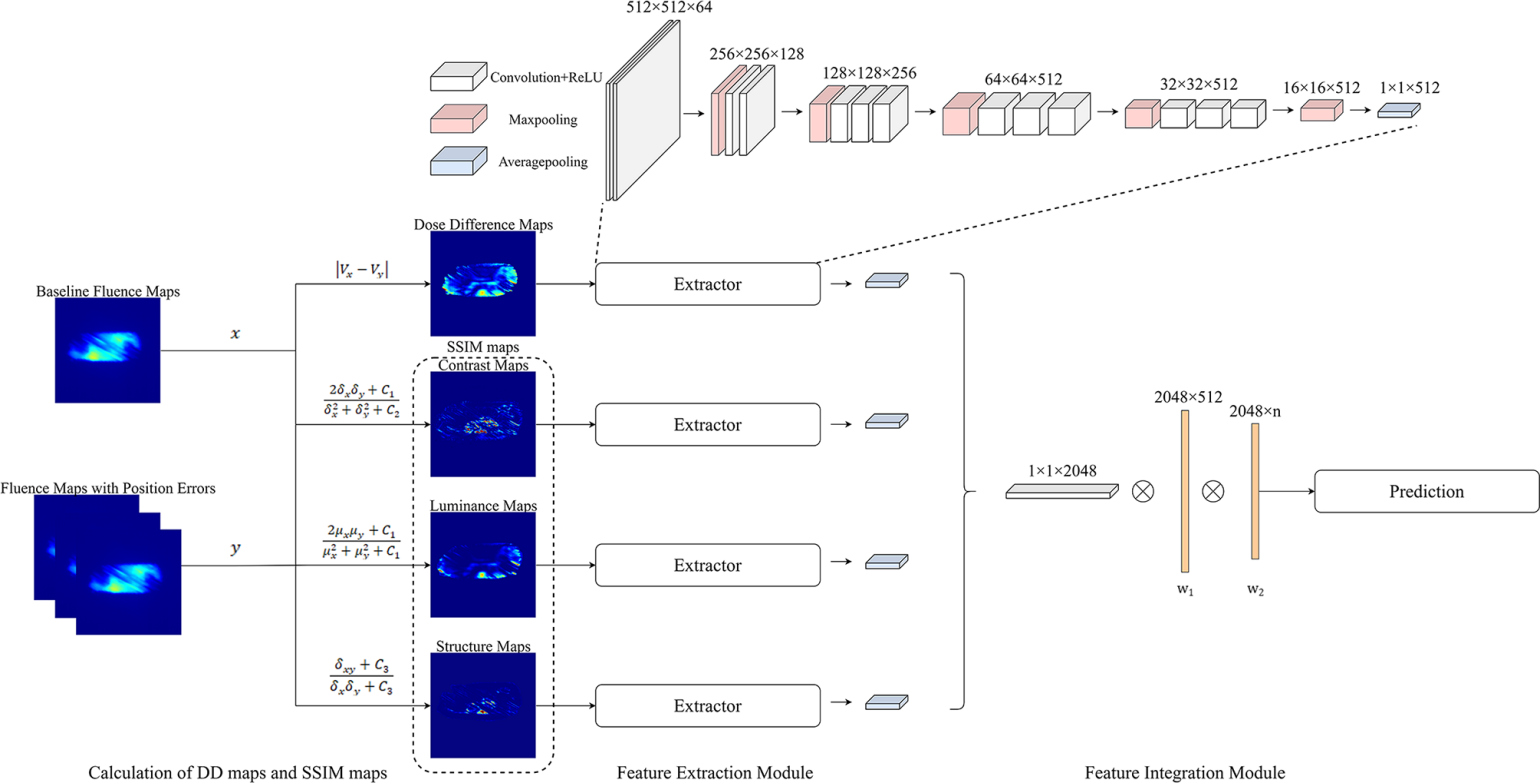
Overview of the process of quantifying the changes in EPID fluence maps and the structure of CNN. The maps with errors are compared with baseline maps to calculate DD maps and SSIM maps. The overall network comprises feature extraction and feature integration modules. Each feature extractor module comprises 13 convolutional layers, four max pooling layers, and one average pooling layer; the feature integration modules comprise two fully connected layers.

Three types of ML models—ML 1 models, which used the radiomics features of DD maps as inputs, ML 2 models, which used the features of SSIM maps as inputs, and ML 3 models, which used the features of DD and SSIM maps as inputs— were used to classify the position errors. Before using the radiomics features for ML, Pearson correlation analysis was performed between the radiomics features and classification labels to exclude features with low correlation and prevent the model from overfitting. To evaluate the performance of each classifier, the data for 32 out of the 40 patients were randomly selected as the training set and the data for the remaining eight patients were used as the testing set. In the training set, leave-one-out cross-validation was combined with a grid search to tune the hyperparameters of the classifiers. The classification labels are listed in **Table 1**.

For the KNN classification, GridSearchCV was used to find a suitable number of neighbors from 1 to 10 with a step of 1. For SVM, GridSearchCV was used to find suitable kernels (kernel = linear, rbf), suitable C (C = 0.01, 0.1, 1, 10), and gamma (gamma = 0.001, 0.01, 0.1, 1) values, with the gamma being used only to find the rbf kernel. For LDC, GridSearchCV was used to find a suitable solver (solver = svd, lsqr, eigen). For XGBoost, 10–100 boosting rounds with a step of 10 and a maximum tree depth of 3–10 were searched for the type 1 and type 2 classification; for the type 3 classification, 100–250 boosting rounds with a step of 10 and a maximum tree depth of 3–10 were searched. The final hyperparameters are shown in **Supplementary Material A**.

### CNN Method

The process flow of the CNN method is illustrated in **Figure 4**. The input data of the CNN method comprised a 512 × 512 center pixel matrix of DD, luminance, contrast, and structure maps. As shown in **Figure 5**, the overall network included a feature extraction module and a feature integration module. In the feature extraction module, four types of maps were input into four independent feature extractors to extract features from different angles. Each extractor comprised 13 convolutional layers, four max pooling layers, and one average pooling layer. The convolutional layers, which did not change the dimension of the data, could extract features of a given dimension deeply, and the rectified linear unit (ReLu) function was used to improve the nonlinear ability of the network. The max pooling layer was used to extract the largest features within a 2 × 2 area and to double the number of channels. The average pooling layer calculated all the eigenvalues of each channel on average. The feature integration module contained two fully connected layers. After the extracted features were integrated, they were each connected to the same latitude within the integration module. The two fully connected layers extracted the relationships between features and output the probabilities of the respective predictions of *n* classes, with the class with the largest predicted value used as the final predicted class. To enable comparison of the ML model and CNN results, the ML training and testing sets were used for the CNN.

### Comparison of ML Models and CNN

The classification accuracies of the ML models and CNN on the test sets were calculated. To evaluate the type 1 and type 2 error classification, the receiver operating characteristic (ROC) curves and the areas under the ROC curve (AUCs) were calculated; to evaluate the type 3 classification, precision, recall, f1-score, and confusion matrices were calculated for the CNN and the best ML 1, 2, and 3 models, respectively. The accuracy, precision, recall, and f1-score are defined as follows:


(7) $$Accuracy=\frac{TP+TN}{TP+TN+FP+FN},$$

(8) $$Precision=\frac{TP}{TP+FP},$$

(9) $$Recall=\frac{TP}{TP+FN},$$

(10) $$F1-score=\frac{2\times Precision\times Recall}{Precision+Recall},$$

where *TP*, *TN*, *FP*, and *FN* denote true positive, true negative, false positive, and false negative, respectively.

## Results

### Classification Results of Machine Learning Method

**Figure 6:**
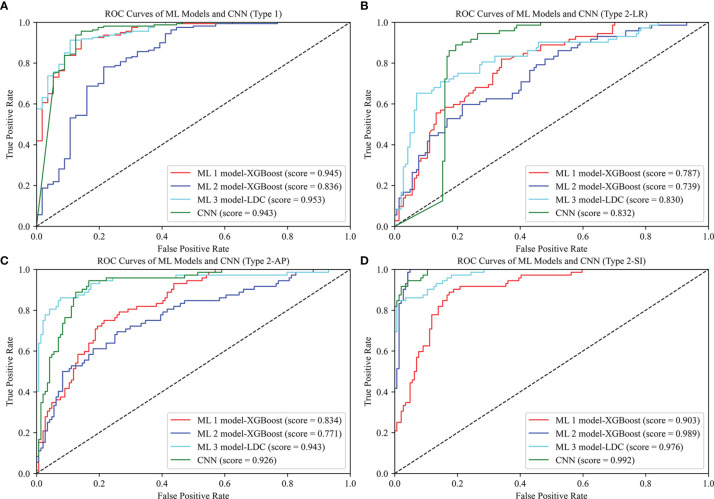
ROC curves of ML 1 model (XGBoost), ML 2 model (XGBoost), ML 3 model (LDC), and CNN. The scores represent the AUC values corresponding to the ROC curves: **(A)** Type 1, **(B)** Type 2-LR, **(C)** Type 2-AP, **(D)** Type 2-SI.

**Figure 7:**
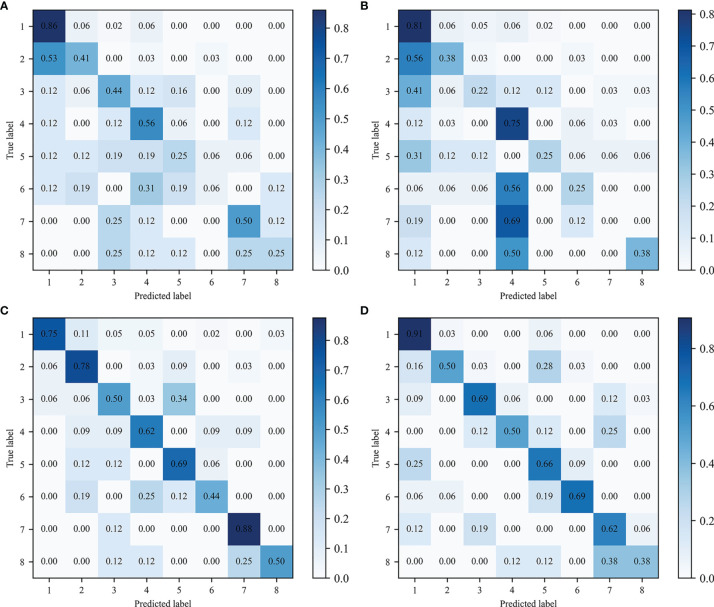
Confusion matrix of type 3 classification. Predicted and true labels 1 to 8 correspond to classes one to eight, respectively: **(A)** ML 1 model-XGBoost, **(B)** ML 2 model- XGBoost, **(C)** ML 3 model-LDC, **(D)** CNN.

As shown in **Table 2**, the best ML 1 model was XGBoost, which had classification accuracies of 0.889, 0.755, 0.778, 0.833, and 0.532 on the type 1, 2-LR, 2-AP, 2-SI, and 3 test sets, respectively. The AUCs for type 1, 2-LR, 2-AP, and 2-SI were 0.945, 0.787, 0.834, and 0.903, respectively (**Figure 6**). As shown in **Table 3** and **Figure 7**, for type 3 errors, the recall value of XGBoost ranged from 0.062 to 0.859, and only the recall values of the first, fourth, and seventh classes were equal to or greater than 0.5.

**Table 2:** Classification accuracy and AUC values of ML 1, 2, and 3 models and CNN.

	Input	Models	Type 1		Type 2-LR		Type 2-AP		Type 2-SI		Type 3	
			Accuracy	AUC	Accuracy	AUC	Accuracy	AUC	Accuracy	AUC	Accuracy	AUC
ML 1 Models	Features of DD maps	LDC	0.852	0.925	0.736	0.817	0.745	0.826	0.782	0.889	0.500	/
		SVM	0.866	0.912	0.755	0.841	0.759	0.778	0.833	0.890	0.519	/
		KNN	0.870	0.914	0.764	0.805	0.736	0.801	0.796	0.889	0.458	/
		XGBoost	0.889	0.945	0.755	0.787	0.778	0.834	0.833	0.903	0.532	/
ML 2 Models	Features of SSIM maps	LDC	0.861	0.892	0.685	0.721	0.750	0.747	0.889	0.975	0.468	/
		SVM	0.787	0.859	0.671	0.722	0.713	0.745	0.884	0.976	0.477	/
		KNN	0.787	0.757	0.657	0.712	0.718	0.682	0.884	0.962	0.324	/
		XGBoost	0.856	0.836	0.731	0.739	0.736	0.771	0.949	0.989	0.491	/
ML 3 Models	Features of DD maps and SSIM maps	LDC	0.903	0.953	0.792	0.830	0.870	0.943	0.931	0.976	0.671	/
		SVM	0.889	0.901	0.778	0.826	0.861	0.928	0.921	0.977	0.662	/
		KNN	0.843	0.906	0.722	0.779	0.722	0.788	0.944	0.990	0.477	/
		XGBoost	0.894	0.934	0.759	0.857	0.759	0.846	0.940	0.986	0.556	/
CNNs	DD maps and SSIM maps	/	0.925	0.943	0.833	0.832	0.875	0.926	0.949	0.992	0.689	/

Because the type 3 classification comprises eight-element classification tasks, the AUC values of this classification type have not been calculated.

**Table 3:** Precision, recall, and f1-score of ML 1 model-XGBoost, ML 2 model-XGBoost, ML 3 model-LDC, and CNN in type 3 classification.

	ML 1 Model - XGBoost			ML 2 Model - XGBoost			ML 3 Model - LDC			CNN		
	Precision	Recall	F1-score	Precision	Recall	F1-score	Precision	Recall	F1-score	Precision	Recall	F1-score
Class 1	0.655	0.859	0.743	0.536	0.812	0.646	0.923	0.750	0.828	0.753	0.906	0.823
Class 2	0.542	0.406	0.464	0.545	0.375	0.444	0.595	0.781	0.676	0.842	0.500	0.627
Class 3	0.500	0.438	0.467	0.500	0.219	0.304	0.593	0.500	0.542	0.786	0.688	0.733
Class 4	0.474	0.562	0.514	0.429	0.750	0.545	0.667	0.625	0.645	0.727	0.500	0.593
Class 5	0.267	0.250	0.258	0.444	0.250	0.320	0.407	0.688	0.512	0.525	0.656	0.583
Class 6	0.333	0.062	0.105	0.400	0.250	0.308	0.583	0.438	0.500	0.733	0.688	0.710
Class 7	0.444	0.500	0.471	0.000	0.000	0.000	0.700	0.875	0.778	0.476	0.625	0.541
Class 8	0.333	0.250	0.286	0.600	0.375	0.462	0.667	0.500	0.571	0.600	0.375	0.462

Precision=TP/(TP+FP) Recall=TP/(TP+FN), and F1-score=(2×Precision×Recall)/(Precision+Recall).

The best ML 2 model was XGBoost, and it obtained accuracies of 0.856, 0.731, 0.736, 0.949, and 0.491 on type 1, 2-LR, 2-AP, 2-SI, and 3 test sets, respectively. The AUCs of type 1, 2-LR, 2-AP, and 2-SI were 0.836, 0.739, 0.771, and 0.989, respectively (**Figure 6**). As shown in **Table 3** and **Figure 7**, for type 3 errors, the recall value of XGBoost ranged from 0.000 to 0.812, and only the recall values of the first and fourth classes exceeded 0.5.

The best ML 3 model was LDC, which obtained accuracies of 0.903, 0.792, 0.870, 0.931, and 0.671 on the type 1, 2-LR, 2-AP, 2-SI, and 3 test sets, respectively. The AUCs of type 1, 2-LR, 2-AP, and 2-SI were 0.953, 0.830, 0.943, and 0.976, respectively (**Figure 6**). As shown in **Table 3** and **Figure 7**, for type 3 errors, the recall value of XGBoost ranged from 0.438 to 0.875, whereas the recall values of all classes were equal to or greater than 0.5, except the sixth class, which had a recall value of 0.438. However, confusions can arise between the third and fifth, fourth and sixth, and seventh and eighth classes.

### Classification Results of CNN Method

As shown in **Table 2**, the CNN achieved classification accuracies of 0.925, 0.833, 0.875, 0.949, and 0.689 on the type 1, 2-LR, 2-AP, 2-SI, and 3 test sets, respectively. The AUCs of the type 1, 2-LR, 2-AP, and 2-SI errors were 0.943, 0.832, 0.926, and 0.992, respectively (**Figure 6**). As shown in **Table 3** and **Figure 7**, for type 3 errors, the recall value ranged from 0.375 to 0.906, with the recall value of all the classes equal to or greater than 0.5, with the exception of the eighth class, for which the recall value is 0.375. The second and fifth, fourth and seventh, and seventh and eighth classes are easy to confuse.

## Discussion

In this study, methods for analyzing EPID transmission fluence maps for the identification of position errors in the treatment of GO patients were developed. Although CBCT is generally used clinically to correct position errors prior to treatment, the dose delivered to a patient cannot be verified using CBCT alone because position or other errors can potentially occur during treatment. To overcome this problem, EPID-based *in vivo* dosimetry has been developed as a monitoring method to help therapists detect problems in a timely manner and enable the accurate treatment of patients. For patients with GO, the rigid anatomical structure around the target volumes ensures that anatomical change has a negligible impact (**Figure 1B**), and the effect of random mechanical error on fluence maps is negligible relative to the effect of position errors. Therefore, this study focused on detecting positional errors in patients with GO.

Most previous studies analyzed EPID fluence maps to detect MLC or MU errors (21, 23, 30). Cecile (32) used CNNs to analyze EPID fluence maps in classifying anatomical changes, position errors, and linac mechanical errors in the treatment of lung cancer patients; however, they used software to simulate fluence maps and applied a 1-cm position error classification boundary. In this study, we measured transmission fluence maps using a linac and classified position errors using a more precise standard; to the best of our knowledge, ours was the first study to classify patient positioning errors using measured EPID transmission fluence maps.

The method of calculating DD is often used clinically to compare dose maps; in this study, the radiomics features of DD maps were used as inputs for the ML 1 models. For type 1 and type 2 classification, the accuracy of XGBoost exceeded 0.75 and the AUCs all exceeded 0.78; however, for type 3 classification, the accuracy was only 0.532 which was unsatisfactory. Meanwhile, for XGBoost in ML 2 models, which used the features of SSIM maps as input, the accuracy and AUCs exceeded 0.73 in type 1 and 2 classification, whereas in type 3 classification, the accuracy was 0.491, which also needed improvement. Combining the DD map features with those of luminance, contrast, and structure maps to classify position errors improved both the accuracies and AUC values; for the type 1 and 2 classification, the accuracy of LDC exceeded 0.79 and the AUCs all exceeded 0.83. A considerable improvement in accuracy was also achieved for the type 3 classification, with the recall value of all but one class equal to or greater than 0.5. These results suggest the usefulness of combining SSIM maps with DD maps in the analysis and detection of errors in EPID transmission fluence maps. SSIM, which can measure the similarities between pairs of images, has been previously investigated as a tool for analyzing EPID fluence maps. Peng Jiayuan et al. demonstrated that the three components of an SSIM map—the luminance, contrast, and structure maps—are capable of detecting the absolute dose error, gradient discrepancy, and dose structure error on two dose planes (22). The use of DD maps can quantify the pixelwise relative differences between baseline maps and maps with errors to compensate for the insensitivity of luminance maps to small luminance differences between images (23).

**Figure 8:**
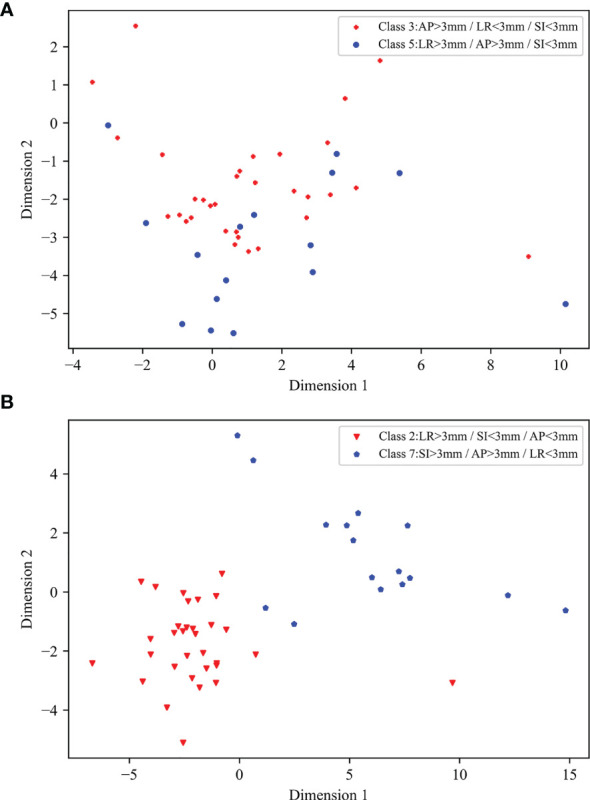
Two-dimensional scatter plots of input data of ML 3 models following linear discriminant analysis dimensionality reduction: **(A)** scatter plot of third and fifth classes, **(B)** scatter plot of second and seventh classes.

The type 1 classification, which initially detects the presence of significant position errors and prompts the therapist and physicist to determine the error in time, was the simplest approach used in this study; the type 2 and type 3 classifications, in contrast, were designed to detect the direction of position errors. The type 3 classification is essentially a combination of the three classification types of the type 2 classification. For the ML 1 and ML 3 models XGBoost and LDC, respectively, simply combining the type 2 classification approaches to conduct type 3 classification produced accuracies of only 0.489 and 0.641, which were both lower than the accuracies of 0.532 and 0.671 achieved by the corresponding type 3 approaches. Therefore, it was necessary to train models specifically for the type 3 classification. All the radiotherapy plans in this study used coplanar VMAT with partial arcs rotating from 240 to 120° clockwise and from 120 to 240° counterclockwise (**Figures 1C–E**), and position errors in the SI direction were perpendicular to the gantry rotation plane. Therefore, the final EPID transmission fluence map integrated the fluence maps from each angle, which contained information on SI direction errors; by contrast, the LR and AP errors all lay on the linac rotational plane and, as a result, the final fluence maps contained less information on LR and AP direction position errors. In addition, the final fluence maps did not contain information covering the gantry angle ranges from 181 to 240° and from 120 to 180°, which could have been used to help classify errors in the LR direction; as a result, in the type 2 classification, the accuracy along the SI direction was the highest whereas that along the LR direction was the lowest. The results of the EPID-based 3D *in vivo* dosimetry study of Li Yinghui also revealed that the SI position error had the most significant impact on the γ pass rate (40). For the type 3 classification, several classes—including the third and fifth classes for the ML 3 models and the seventh and eighth classes for the CNN—were easy to confuse. This confusion arose from the fact that both the fifth and eighth classes introduced LR errors, which had a considerably lesser influence on the fluence maps than that of the AP and SI errors introduced by the third and seventh classes. Linear discriminant analysis was used to project the ML 3 model input data onto a two-dimensional scatter plot for visualization (**Figure 8**), from which it was observed that the data for classes that were easily confused (such as the third and fifth classes) overlapped (**Figure 8** **A**) and were more difficult to classify relative to the data for easier-to-distinguish classes (**Figure 8** **B**), such as the second and seventh classes.

The EPID dosimetry study conducted by Ma Chaoqiong (23) used LDC, SVM, and random forest (RF) methods to train models, whereas Nyflot Matthew J (30) used SVM, KNN, and decision tree (DT) approaches. In this study, LDC, SVM, and KNN were used and XGBoost was used instead of RF or DT in training the ML models, and the four approaches were found to have equivalent classification power. Although the performance of KNN in the type 1 and type 2 classifications was found to be acceptable, the accuracies of KNN in the ML 1, 2, and 3 models for the type 3 classification were sub-optimal at 0.458, 0.324, and 0.477, respectively. These low accuracies may be attributed to the unbalanced distribution of sample sizes among the eight categories, as KNN is prone to classification errors in situations involving imbalances in sample size (41). The ML 1 and 2 model achieved relatively good results using XGBoost; for the ML 3 model, by contrast, XGBoost was not as effective as LDC because the dimensionality of the ML 3 model input data was higher than that of the ML 1 and 2 model data and XGBoost is prone to overfitting when processing high-dimensional feature data (42). The CNN achieved a slight improvement in classification accuracy relative to that of the ML 3 model. The two models also produced similar AUC values. In this study, for each patient, 28 fluence maps were measured, and there were 1120 fluence maps in total. After classification, there were less maps corresponding to each label, the size of the data set (40 patients) used as CNN input may have been insufficient for the network, as CNNs generally require large-scale datasets for training (43, 44); as a result, the advantage of using the CNNs was not obvious.

In this study, a method to identify position errors was developed by analyzing the EPID fluence maps, which can be combined with CBCT in clinical treatment. At the first few fractions, fluence maps should be acquired after using CBCT to correct the position errors. The maps of the first fraction should be used as baseline, and a comparison should be conducted between the baseline maps and other fluence maps. If the results show no obvious errors, the frequency of using CBCT should be reduced, and the method of using fluence maps to identify position errors should be used to monitor the next fractions. However, small position errors such as 1 mm errors can be more common in clinical, therefore the feasibility of this monitoring process needs to be further studied. All the EPID transmission fluence maps in this study were measured on the same day for each patient plan and were based on the Chengdu dosimetric phantoms using as1200 EPID; however, deviation of absolute dose, flatness, and symmetry should be considered when monitoring the patients clinically. Meanwhile, the impact brought by different resolution of different EPID detectors and different anatomical structures of different patients on the method we proposed in this study requires further exploration. In addition, the fluence map analysis was limited to the identification of translational position errors in the treatment of GO patients. Study of Cecile (32) has shown different types of errors such as rotational position errors, anatomical changes, and linac mechanical errors that can be detected by EPID dosimetry for lung cancer patients. Further research involving the use of EPID transmission fluence maps to monitor more types of errors and diseases should be conducted.

## Conclusion

DD and SSIM maps can be combined to analyze EPID transmission fluence maps. ML models as well as CNN trained on small-sized samples can be used to identify position errors in the treatment of GO patients. Further studies with large sample sizes are required to improve the accuracy of CNN. The feasibility of using this method in clinical treatment should be further investigated.

## Data Availability Statement

The original contributions presented in the study are included in the article/**Supplementary Material**. Further inquiries can be directed to the corresponding authors.

## Ethics Statement

The studies involving human participants were reviewed and approved by the ethics committee of the West China Hospital (ChiCTR2100043576). Written informed consent for participation was not required for this study in accordance with the national legislation and the institutional requirements.

## Author Contributions

XZ, GL, and GD contributed conception and design of the study. SB and GL contributed to administrative support and provision of study materials or patients. GD, XZ, ZL, GW, YL, QX, LD, JL, and XS collected the data. WL, GD, XZ, and GL analyzed the data. All authors contributed to the article and approved the submitted version.

## Funding

This work was supported by the National Natural Science Foundation of China (grant numbers 81472807 and 81972848), and the Sichuan Science and Technology Program (grant number 2021YFS0143).

## Conflict of Interest

The authors declare that the research was conducted in the absence of any commercial or financial relationships that could be construed as a potential conflict of interest.

## Publisher’s Note

All claims expressed in this article are solely those of the authors and do not necessarily represent those of their affiliated organizations, or those of the publisher, the editors and the reviewers. Any product that may be evaluated in this article, or claim that may be made by its manufacturer, is not guaranteed or endorsed by the publisher.

## References

[1] Zeng L Xie XQ Li CH Shi HS Wang F. Clinical Study of the Radiotherapy With EDGE Accelerator in the Treatment of the Moderate and Severe Thyroid Associated Ophthalmopathy. Eur Rev Med Pharmacol Sci (2019) 23(8):3471–7. doi: 10.26355/eurrev_201904_17712 31081102

[2] Li YJ Luo Y He WM Li P Wang F. Clinical Outcomes of Graves' Ophthalmopathy Treated With Intensity Modulated Radiation Therapy. Radiat Oncol (2017) 12(1):171. doi: 10.1186/s13014-017-0908-7 29110673 PMC5674803

[3] Wang SC Wu J Xie XQ Liu XL Luo Y Wang F. Comparison of IMRT and VMAT Radiotherapy Planning for Graves' Ophthalmopathy Based on Dosimetric Parameters Analysis. Eur Rev Med Pharmacol Sci (2020) 24(7):3898–906. doi: 10.26355/eurrev_202004_20856 32329865

[4] Park JM Kim K Chie EK Choi CH Ye SJ Ha SW. RapidArc *vs* Intensity-Modulated Radiation Therapy for Hepatocellular Carcinoma: A Comparative Planning Study. Br J Radiol (2012) 85(1015):e323–9. doi: 10.1259/bjr/19088580 PMC3474061 22745211

[5] Otto K. Volumetric Modulated Arc Therapy: IMRT in a Single Gantry Arc. Med Phys (2008) 35(1):310–7. doi: 10.1118/1.2818738 18293586

[6] Kim JI Park SY Kim HJ Kim JH Ye SJ Park JM. The Sensitivity of Gamma-Index Method to the Positioning Errors of High-Definition MLC in Patient-Specific VMAT QA for SBRT. Radiat Oncol (2014) 9:167. doi: 10.1186/1748-717X-9-167 25070065 PMC4118611

[7] Alaei P Spezi E. Imaging Dose From Cone Beam Computed Tomography in Radiation Therapy. Phys Med (2015) 31(7):647–58. doi: 10.1016/j.ejmp.2015.06.003 26148865

[8] Pang PP Hendry J Cheah SL Soong YL Fong KW Wee TS. An Assessment of the Magnitude of Intra-Fraction Movement of Head-and-Neck IMRT Cases and Its Implication on the Action-Level of the Imaging Protocol. Radiother Oncol (2014) 112(3):437–41. doi: 10.1016/j.radonc.2014.09.008 25284062

[9] Heijkoop ST Langerak TR Quint S Mens JW Zolnay AG Heijmen BJ. Quantification of Intra-Fraction Changes During Radiotherapy of Cervical Cancer Assessed With Pre- and Post-Fraction Cone Beam CT Scans. Radiother Oncol (2015) 117(3):536–41. doi: 10.1016/j.radonc.2015.08.034 26409830

[10] Apicella G Loi G Torrente S Crespi S Beldi D Brambilla M. Three-Dimensional Surface Imaging for Detection of Intra-Fraction Setup Variations During Radiotherapy of Pelvic Tumors. Radiol Med (2016) 121(10):805–10. doi: 10.1007/s11547-016-0659-9 27300649

[11] Vial P Gustafsson H Oliver L Baldock C Greer PB. Direct-Detection EPID Dosimetry: Investigation of a Potential Clinical Configuration for IMRT Verification. Phys Med Biol (2009) 54(23):7151–69. doi: 10.1088/0031-9155/54/23/008 19904032

[12] van Elmpt W McDermott L Nijsten S Wendling M Lambin P Mijnheer B. A Literature Review of Electronic Portal Imaging for Radiotherapy Dosimetry. Radiother Oncol (2008) 88(3):289–309. doi: 10.1016/j.radonc.2008.07.008 18706727

[13] Van Esch A Depuydt T Huyskens DP. The Use of an aSi-Based EPID for Routine Absolute Dosimetric Pre-Treatment Verification of Dynamic IMRT Fields. Radiother Oncol (2004) 71(2):223–34. doi: 10.1016/j.radonc.2004.02.018 15110457

[14] Deshpande S Blake SJ Xing A Metcalfe PE Holloway LC Vial P. A Simple Model for Transit Dosimetry Based on a Water Equivalent EPID. Med Phys (2018) 45(3):1266–75. doi: 10.1002/mp.12742 29314080

[15] Martinez Ortega J Gomez Gonzalez N Castro Tejero P Pinto Monedero M Tolani NB Nunez Martin L. A Portal Dosimetry Dose Prediction Method Based on Collapsed Cone Algorithm Using the Clinical Beam Model. Med Phys (2017) 44(1):333–41. doi: 10.1002/mp.12018 28102946

[16] Kang S Li J Ma J Zhang W Liao X Qing H. Evaluation of Interfraction Setup Variations for Postmastectomy Radiation Therapy Using EPID-Based *In Vivo* Dosimetry. J Appl Clin Med Phys (2019) 20(10):43–52. doi: 10.1002/acm2.12712 PMC6806484 31541537

[17] Ahmed S Kapatoes J Zhang G Moros EG Feygelman V. A Hybrid Volumetric Dose Verification Method for Single-Isocenter Multiple-Target Cranial SRS. J Appl Clin Med Phys (2018) 19(5):651–8. doi: 10.1002/acm2.12430 PMC6123151 30112817

[18] Zhuang AH Olch AJ. Sensitivity Study of an Automated System for Daily Patient QA Using EPID Exit Dose Images. J Appl Clin Med Phys (2018) 19(3):114–24. doi: 10.1002/acm2.12303 PMC5978566 29508529

[19] Moliner G Sorro L Verstraet R Daviau PA Casas M Piron B. Assessment of Combined Use of ArcCheck((R)) Detector and Portal Dosimetry for Delivery Quality Assurance of Head and Neck and Prostate Volumetric-Modulated Arc Therapy. J Appl Clin Med Phys (2018) 19(6):133–9. doi: 10.1002/acm2.12460 PMC6236827 30338922

[20] Hsieh ES Hansen KS Kent MS Saini S Dieterich S. Can a Commercially Available EPID Dosimetry System Detect Small Daily Patient Setup Errors for Cranial IMRT/SRS? Pract Radiat Oncol (2017) 7(4):e283–e90. doi: 10.1016/j.prro.2016.12.005 28336480

[21] Wootton LS Nyflot MJ Chaovalitwongse WA Ford E. Error Detection in Intensity-Modulated Radiation Therapy Quality Assurance Using Radiomic Analysis of Gamma Distributions. Int J Radiat Oncol Biol Phys (2018) 102(1):219–28. doi: 10.1016/j.ijrobp.2018.05.033 30102197

[22] Peng J Shi C Laugeman E Hu W Zhang Z Mutic S. Implementation of the Structural SIMilarity (SSIM) Index as a Quantitative Evaluation Tool for Dose Distribution Error Detection. Med Phys (2020) 47(4):1907–19. doi: 10.1002/mp.14010 PMC7472642 31901143

[23] Ma C Wang R Zhou S Wang M Yue H Zhang Y. The Structural Similarity Index for IMRT Quality Assurance: Radiomics-Based Error Classification. Med Phys (2020) 48(1):80–93. doi: 10.1002/mp.14559 33128263

[24] Gillies RJ Kinahan PE Hricak H. Radiomics: Images Are More Than Pictures, They Are Data. Radiology (2016) 278(2):563–77. doi: 10.1148/radiol.2015151169 PMC4734157 26579733

[25] Lambin P Leijenaar RTH Deist TM Peerlings J de Jong EEC van Timmeren J. Radiomics: The Bridge Between Medical Imaging and Personalized Medicine. Nat Rev Clin Oncol (2017) 14(12):749–62. doi: 10.1038/nrclinonc.2017.141 28975929

[26] Yu J Deng Y Liu T Zhou J Jia X Xiao T. Lymph Node Metastasis Prediction of Papillary Thyroid Carcinoma Based on Transfer Learning Radiomics. Nat Commun (2020) 11(1):4807. doi: 10.1038/s41467-020-18497-3 32968067 PMC7511309

[27] Mu W Jiang L Zhang J Shi Y Gray JE Tunali I. Non-Invasive Decision Support for NSCLC Treatment Using PET/CT Radiomics. Nat Commun (2020) 11(1):5228. doi: 10.1038/s41467-020-19116-x 33067442 PMC7567795

[28] Conti A Duggento A Indovina I Guerrisi M Toschi N. Radiomics in Breast Cancer Classification and Prediction. Semin Cancer Biol (2020) 72:238–50. doi: 10.1016/j.semcancer.2020.04.002 32371013

[29] Cai J Zheng J Shen J Yuan Z Xie M Gao M. A Radiomics Model for Predicting the Response to Bevacizumab in Brain Necrosis After Radiotherapy. Clin Cancer Res (2020) 26(20):5438–47. doi: 10.1158/1078-0432.CCR-20-1264 32727886

[30] Nyflot MJ Thammasorn P Wootton LS Ford EC Chaovalitwongse WA. Deep Learning for Patient-Specific Quality Assurance: Identifying Errors in Radiotherapy Delivery by Radiomic Analysis of Gamma Images With Convolutional Neural Networks. Med Phys (2019) 46(2):456–64. doi: 10.1002/mp.13338 30548601

[31] Shen C Nguyen D Zhou Z Jiang SB Dong B Jia X. An Introduction to Deep Learning in Medical Physics: Advantages, Potential, and Challenges. Phys Med Biol (2020) 65(5):05TR1. doi: 10.1088/1361-6560/ab6f51 PMC7101509 31972556

[32] Wolfs CJA Canters RAM Verhaegen F. Identification of Treatment Error Types for Lung Cancer Patients Using Convolutional Neural Networks and EPID Dosimetry. Radiother Oncol (2020) 153:249–9. doi: 10.1016/j.radonc.2020.09.048 33011206

[33] Tomori S Kadoya N Takayama Y Kajikawa T Shima K Narazaki K. A Deep Learning-Based Prediction Model for Gamma Evaluation in Patient-Specific Quality Assurance. Med Phys (2018) 45(9):4055–65. doi: 10.1002/mp.13112 30066388

[34] Potter NJ Mund K Andreozzi JM Li JG Liu C Yan G. Error Detection and Classification in Patient-Specific IMRT QA With Dual Neural Networks. Med Phys (2020) 47(10):4711–20. doi: 10.1002/mp.14416 33460182

[35] Kimura Y Kadoya N Tomori S Oku Y Jingu K. Error Detection Using a Convolutional Neural Network With Dose Difference Maps in Patient-Specific Quality Assurance for Volumetric Modulated Arc Therapy. Phys Med (2020) 73:57–64. doi: 10.1016/j.ejmp.2020.03.022 32330812

[36] Wang Z Bovik AC Sheikh HR Simoncelli EP. Image Quality Assessment: From Error Visibility to Structural Similarity. IEEE Trans Image Process (2004) 13(4):600–12. doi: 10.1109/tip.2003.819861 15376593

[37] van Griethuysen JJM Fedorov A Parmar C Hosny A Aucoin N Narayan V. Computational Radiomics System to Decode the Radiographic Phenotype. Cancer Res (2017) 77(21):e104–7. doi: 10.1158/0008-5472.CAN-17-0339 PMC5672828 29092951

[38] Pedregosa F Varoquaux G Gramfort A Michel V Thirion B Grisel O. Scikit-Learn: Machine Learning in Python. J Mach Learn Res (2011) 12:2825–30.

[39] Chen TQ Guestrin C Assoc Comp M. XGBoost: A Scalable Tree Boosting System. New York: Assoc Computing Machinery (2016). p. 785–94.

[40] Li Y Zhu J Shi J Chen L Liu X. Investigating the Effectiveness of Monitoring Relevant Variations During IMRT and VMAT Treatments by EPID-Based 3D *In Vivo* Verification Performed Using Planning CTs. PloS One (2019) 14(6):e0218803. doi: 10.1371/journal.pone.0218803 31251751 PMC6599132

[41] Sun J Du W Shi NJ. A Survey of kNN Algorithm .Open J Syst (2018) 1(1):1–10. doi: 10.18063/ieac.v1i1.770

[42] Li WT Ma J Shende N Castaneda G Chakladar J Tsai JC. Using Machine Learning of Clinical Data to Diagnose COVID-19: A Systematic Review and Meta-Analysis. BMC Med Inform Decis Mak (2020) 20(1):247. doi: 10.1186/s12911-020-01266-z 32993652 PMC7522928

[43] Wong GL Yuen PC Ma AJ Chan AW Leung HH Wong VW. Artificial Intelligence in Prediction of Non-Alcoholic Fatty Liver Disease and Fibrosis. J Gastroenterol Hepatol (2021) 36(3):543–50. doi: 10.1111/jgh.15385 33709607

[44] Liu H Jiao Z Han W Jing B. Identifying the Histologic Subtypes of Non-Small Cell Lung Cancer With Computed Tomography Imaging: A Comparative Study of Capsule Net, Convolutional Neural Network, and Radiomics. Quant Imaging Med Surg (2021) 11(6):2756–65. doi: 10.21037/qims-20-734 PMC8107316 34079739

