# Supplementary material for: Analysis of EPID Transmission Fluence Maps Using Machine Learning Models and CNN for Identifying Position Errors in the Treatment of GO Patients
Source: Front Oncol. 2021 Sep 14;11:721591. doi: 10.3389/fonc.2021.721591 (PMC8476908; doi:10.3389/fonc.2021.721591)
Supplement: Supplementary file 1 [file DataSheet_1.docx]

Supplementary Material A Hyperparameters of ML 1 models, ML 2 models, and ML 3 models.

|  | Models | Type 1 | Type 2 | | | Type 3 |
| --- | --- | --- | --- | --- | --- | --- |
|  |  |  | LR | AP | SI |  |
| ML 1 models | XGBoost | n_estimators=70  max_depth=7 | n_estimators=30  max_depth=4 | n_estimators=140  max_depth=9 | n_estimators=130  max_depth=6 | n_estimators=180  max_depth=3 |
|  | KNN | n_neighbors=5 | n_neighbors=10 | n_neighbors=9 | n_neighbors=8 | n_neighbors=5 |
|  | SVM | Kernel=RBF  C=10  Gamma=0.1 | Kernel=RBF  C=10  Gamma=0.1 | Kernel=RBF  C=10  Gamma=0.1 | Kernel=RBF  C=1  Gamma=0.1 | Kernel=RBF  C=10  Gamma=0.01 |
|  | LDC | Solver=svd | Solver=svd | Solver=svd | Solver=svd | Solver=svd |
| ML 2 models | XGBoost | n_estimators=70  max_depth=9 | n_estimators=100  max_depth=10 | n_estimators=130  max_depth=5 | n_estimators=100  max_depth=3 | n_estimators=140  max_depth=3 |
|  | KNN | n_neighbors=8 | n_neighbors=10 | n_neighbors=9 | n_neighbors=9 | n_neighbors=6 |
|  | SVM | Kernel=RBF  C=10  Gamma=0.001 | Kernel=RBF  C=10  Gamma=0.1 | Kernel=RBF  C=10  Gamma=0.01 | Kernel=Linear  C=10 | Kernel=Linear  C=10 |
|  | LDC | Solver=svd | Solver=svd | Solver=svd | Solver=svd | Solver=svd |
| ML 3 models | XGBoost | n_estimators=90  max_depth=7 | n_estimators=130  max_depth=3 | n_estimators=130  max_depth=3 | n_estimators=100  max_depth=10 | n_estimators=240  max_depth=3 |
|  | KNN | n_neighbors=5 | n_neighbors=6 | n_neighbors=10 | n_neighbors=10 | n_neighbors=9 |
|  | SVM | Kernel=RBF  C=10  Gamma=0.1 | Kernel=Linear  C=10 | Kernel=RBF  C=10  Gamma=0.01 | Kernel=Linear  C=0.1 | Kernel=RBF  C=10  Gamma=0.01 |
|  | LDC | Solver=svd | Solver=svd | Solver=svd | Solver=svd | Solver=svd |
